# Cardioprotective effect of antiviral therapy among hepatitis C infected patients: A meta-analysis

**DOI:** 10.1016/j.ijcha.2023.101270

**Published:** 2023-09-22

**Authors:** Vikash Jaiswal, Song Peng Ang, Muhammad Hanif, Mayank Jha, Vikash Kumar, Abdelmonem Siddiq, Bhavyakumar Vachhani, Anupam Halder, Michelle Koifman, Herby Jeanty, Siddharath Soni, Madeeha Subhan Waleed, Tushar Kumar, Helen Huang, Dhrubajyoti Bandyopadhyay

**Affiliations:** a Department of Research, Larkin Community Hospital, USA; b JCCR Cardiology Research, Varanasi, India; c Department of Internal Medicine, Rutgers Health/Community Medical Center, NJ, USA; d Department of Internal Medicine, SUNY Upstate Medical University, Syracuse, NY, USA; e Department of Medicine and Surgery, Government Medical College, Surat, India; f Department of Internal Medicine, The Brooklyn Hospital Center, NY, USA; g Department of Pharmacy, Mansoura University, Mansoura 35516, Egypt; h Department of Internal Medicine, UPMC Harrisburg, PA, USA; i Shree Narayan Medical Institute and Hospital, Saharsa, Bihar, India; j Department of Internal Medicine, Lower Bucks Hospital, Bristol, USA; k Department of Radiology, Sikkim Manipal Institute of Medical Science, Gangtok, India; l RCSI University of Medicine and Health Sciences, Dublin, Ireland; m Department of Cardiology, New York Medical College at Westchester Medical Center, NY, USA

**Keywords:** Hepatitis C, Antiviral therapy, Cardiovascular diseases, Outcomes

## Abstract

**Background:**

Hepatitis C (HCV) infections have been shown to be associated a with higher risk of atherosclerotic cardiovascular disease (CVD). However, the use of antiviral therapy (AVT) and the risk of CVD has not been well established with limited literature.

**Objective:**

We sought to evaluate the association between AVT use post-HCV infection and cardiovascular outcomes.

**Methods:**

We performed a systematic literature search using PubMed, Embase, and Scopus for relevant articles from inception until 10th March 2023. Primary clinical outcomes were the incidence of any CVD. Secondary endpoints were all-cause of mortality, stroke, myocardial infarction, and peripheral artery disease.

**Results:**

A total of 394,452 patients were included in the analysis (111,076 in the AVT group and 283,376 patients in the NAVT group). The mean age of patients among AVT and NAVT groups was comparable (58.7 vs 58.18). The pooled analysis of primary outcomes showed that AVT was associated with a significantly reduced risk of any CVD (HR, 0.55(95%CI: 0.41–0.75), P < 0.001) compared with the NAVT group of patients. Secondary outcomes including ACM (HR, 0.38(95%CI: 0.32–0.46), P < 0.001), MI (HR, 0.62(95%CI: 0.41–0.94), P = 0.02), and PAD (HR, 0.62(95%CI: 0.41–0.93), P = 0.02) were significantly lower among AVT groups compared with NAVT groups of patients with HCV infection. However, the risk of stroke was comparable between both groups of patients (HR, 0.79(95%CI: 0.58–1.07), P = 0.13).

**Conclusion:**

Our analysis shows HCV-infected patients post-AVT have a significantly lower risk of any CVD, MI, ACM, and PAD compared with NAVT groups of patients.

## Highlights

• Limited studies with conflicting results on antiviral therapy among HCV infected patients and its effect on cardiovascular outcomes is available.
• This meta-analysis with the highest sample size shows that HCV-infected patients post-AVT have a significantly lower risk of any CVD, MI, ACM, and PAD compared with NAVT groups of patients.
• The cardioprotective effect of this AVT can be helpful in reducing adverse cardiovascular outcomes and mortality associated with it.

## 1 Introduction

Hepatitis C is a viral disease caused by the hepatitis C virus (HCV), which is a blood-borne virus usually transmitted from unsafe injection practices [1]. Seventy percent of the patients infected with HCV develop a chronic disease, while the rest experience acute transient infection. It was reported that around 58 million people are infected with HCV globally with an estimated incidence of 1.5 million per year [2]. Chronic HCV is associated with chronic inflammation and damage in the liver tissue causing progressive stages of fibrosis and cirrhosis, which are considered pre-carcinogenic stages before the development of hepatocellular carcinoma (HCC). It was estimated that the risk for HCC development increases 17-fold in HCV-infected patients [3], [4]. Chronic HCV infection contributes to the development of insulin resistance, steatohepatitis, and modification in the lipid metabolism and inflammatory signals causing a group of liver non-related events (Extrahepatic manifestations). Extrahepatic manifestations such as CVD (Stroke and ischemic heart disease), renal diseases, central nervous system complications, metabolic syndrome, and type 2 diabetes mellitus [5], [6], [7], [8].

Several studies have reported a significant association between HCV infection and CVD, such as myocardial infarction, coronary heart disease, myocardial injury, and cardiac arrhythmias [9], [10], [11], [12]. Several meta-analyses have reported that HCV infection is associated with carotid atherosclerosis, an increment in cardiovascular morbidity and mortality, and an increase in stroke development risk [13], [14], [15], [16]. Adinolfi LE et al. reported that the prevalence of HCV infection in ischemic stroke was significantly higher than that in controls (26.8% vs. 6.6%) [16]. In another retrospective cohort study, HCV infection was found to be an independent predictor of stroke and cerebrovascular disease [15]. Similarly, a higher risk of peripheral artery disease (PAD) development and carotid artery stenosis was reported in HCV-infected patients as compared to non-HCV-infected control [17], [18]. Several cohort studies have reported that HCV infection was associated with increased cardiovascular mortality, and this was supported by a meta-analysis conducted by Ken et al. which found that the burden of CVD associated with HCV infection equals 1.5 million Disability-adjusted life years (DALYs) per year with the highest burden in the low and middle-income countries [19], [20], [21], [22]. Several antiviral therapies (AVT) have been used to decrease the global burden of HCV with the major aim of achieving a sustained virologic response (SVR) to curb the progression of the disease, prevent cirrhosis development, and decrease the risk of HCC development [23].

Achieving SVR was reported to be associated with a decrease in liver-related and unrelated events, HCC incidence, and overall mortality [24]. In a cohort study conducted among 1599 patients, 39% of them achieved SVR after the treatment with interferon plus ribavirin which was associated with a decrease in the non-liver related events [25]. Direct-acting AVT have been reported as an effective and safe substitute for interferons and were reported to be associated with the achievement of SVR in up to 99% of HCV patients [26]. The achievement of SVR by direct-acting AVT was reported to be associated with a decrease in the risk of extrahepatic manifestations [13], [27], [28].

The effect of these antiviral therapies on cardiovascular outcomes has been discussed in several studies with conflicting results [29], [30], [31]. So we aim to evaluate the association between AVT and cardiovascular outcomes and mortality among HCV infected patients.

## 2 Methods

### 2.1 Materials and methods

This meta-analysis was conducted and reported following the Cochrane and PRISMA (Preferred reporting items for systematic review and Meta-analysis) [32] and MOOSE (Meta-analysis of Observational Studies in Epidemiology) [33] guidelines and performed according to established methods, as described previously [34], [35], [36]. The pre-specified study protocol has been registered in the PROSPERO (**CRD42023410973)**.

### 2.2 Search strategy

We conducted a systematic literature search in PubMed, Embase, and Scopus using predefined MESH terms by using “AND” and “OR”. The following search terms were used: (((((((((hepatitis c[MeSH Terms]) OR (chronic hepatitis c[MeSH Terms])) AND (antiviral agents[MeSH Terms])) OR (antiviral therapy[Other Term])) AND (myocardial infarction[MeSH Terms])) OR (acute coronary syndrome[MeSH Terms])) AND (acute stroke[MeSH Terms])) AND (disease, peripheral artery[MeSH Terms])) AND (mortality[Other Term])) AND (heart failure[MeSH Terms]). The search was performed from inception up until 10th March 2023 without any restrictions on the language of the studies.

All the studies were carefully screened and exported to the Mendeley reference manager used to handle searched citations. A manual check was carried through to crosscheck for any remaining duplicates. Two reviewers (VJ and MJ) reviewed the papers based on the title and abstract. Discrepancies regarding the inclusion of studies were arbitrated by another author (MH).

### 2.3 Eligibility criteria

We included studies with adult patients ≥18 years of age with all patients with Hepatitis C infection. All prospective and retrospective cohort studies were sought to be eligible for inclusion in the study. It was decided to include studies with two arms in order to make a comparison between patients with AVT as the intervention arm, and NAVT as a comparator group post-HCV. Studies were sought to be eligible if they have given cardiovascular outcomes of interest. Selected studies compared patients with varying baseline characteristics and pathologies along with data for outcomes of interest.

Studies that were performed on animals, or reviews, case reports, case series, studies on patients <18 years, studies with a single arm or without HCV infection or without AVT, and studies without outcomes of interest were excluded from the review.

### 2.4 Clinical outcomes

The primary outcome of this meta-analysis was the risk of any cardiovascular disease. The secondary outcomes were incidences of Stroke, Peripheral artery disease, Myocardial infarction, and All-cause mortality.

### 2.5 Data extraction and quality assessment

Two authors (MJ and VJ) extracted the following data: study type, author, study location, study follow-up duration, patient characteristics (number, age, gender, and comorbidities), and primary and secondary outcomes**.** We used the reported estimates when reported in the form of hazard ratios (HRs). If different estimates were available, we would have opted for HR with the most adjusted effect measure. Two investigators (MJ and VJ) independently appraised the potential risk of bias using the Newcastle-Ottawa (NOS) scale for observational studies. [37] We then classified studies as low, moderate, or high quality based on the scores after evaluation.

### 2.6 Statistical analysis

Statistical analysis was performed by calculating the hazard ratio (HR) using the random effect model, with a test for overall effect reported as Z-value, 95% confidence interval (CI), and probability value (P) [38]. Statistical significance was met if 95% CI does not cross numeric “1″ and p < 0.05. The heterogeneity among studies was assessed by Higgins's I-squared (I^2^) statistical model with I^2^ values. As a guide, I^2^ < 25% indicated low, 25–50% moderate, and >50% high heterogeneity [39]. Further sensitivity analyses were performed using a leave-one-out method to check the robustness of the results. Assessment of publication bias was via visualization of the funnel plot. If publication bias is detected, the trim-and-fill method will be employed to adjust for publication bias. All statistical work, including analyses and graphical illustrations, was conducted using STATA (version 17.0, StataCorp) [40].

## 3 Results

### 3.1 Study selection

The preliminary database search using the pre-specified keywords yielded 974 articles, of which 175 studies were excluded after the removal of duplicates. 778 studies were further excluded from the initial post-title and abstract screening based on the inclusion and exclusion criteria and comparison arm (AVT vs. NAVT). The full-text review was conducted for the remaining 21 articles identified during the search period, of which 13 studies were excluded: non hepatitis infected patients or with desired outcomes, single arm or abstract without any desired outcome. Hence, a total of 8 studies [41], [42], [43], [27], [31], [44], [29], [45] met the eligibility criteria and were included in the meta-analysis. The Preferred Reporting Items for Systematic Reviews and Meta-Analyses (PRISMA) flow diagram is depicted in Supplementary Fig. 1**.**

### 3.2 Baseline patient demographics

A total of 394,452 patients with Hepatitis C infection were included into the analysis (111,076 in the AVT group and 283,376 patients in the NAVT group). The mean age of patients among AVT and NAVT groups was comparable (58.7 vs 58.18). The most common comorbidity among AVT and NAVT groups was hypertension (31% vs 29%), and diabetes mellitus (17% vs 22%). The study characteristics, demographics, and comorbidities are presented in Table 1**.**

**Table 1:** Baseline demographics, comorbidities, and study characteristics of studies included in the meta-analysis.

**Authors**	**Sample Size, n**	**Study design**	**Men, %**	**Mean age, year**	**Therapy regime**	**DM, %**	**HTN, %**	**Followup, years**
**Lam et al**	8,148	Prospective Cohort	53.6	56.2/54.1	DAA	12/9	28/26	4.29/4.51 years
**McGlynn et al**	33,808	Retrospective Cohort	61.8	57.2	DAA	–	–	6 months
**Butt et al**	34,206	Retrospective Cohort	96.1	59/58	DAA/INF	8.4/9.3	51/54.1	–
**Adinolfi et al**	2249	Prospective Cohort	48	71/70	DAA	18.7/16.2	44.8/45.5	28 months
**Lin et al**	32,568	Retrospective Cohort	55.6	49.8/49.7	INF	8.1/7.7	14.9/14.5	4.4/4.3 years
**Sasso et al**	770	Prospective Cohort	46.7	69/68	DAA	–	51.9/49.2	25/22 months
**Hsu et al**	37,152	Prospective Cohort	58.9	51.8/51.7	INF	13.9/13.9	21.6/21.7	3.3/3.2 years
**Ogawa et al**	2,45,596	Retrospective Cohort	58.9	59.9/58.5	DAA	26.3/25.4	–	–

*DM: Diabetes mellitus; HTN: Hypertension.

### 3.3 Meta-analysis of clinical outcomes among patients with Hepatitis C infection

**Fig. 1:**
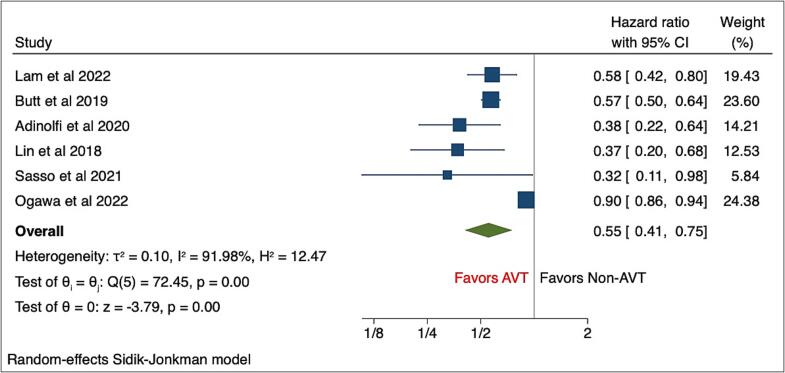
Forest plots of primary outcome - Any CVD.

The pooled analysis of primary outcomes showed that AVT was associated with a reduced risk of any CVD (HR, 0.55(95%CI: 0.41–0.75), P < 0.001) compared with the NAVT group of patients **(**Fig. 1**)**.

**Fig. 2:**
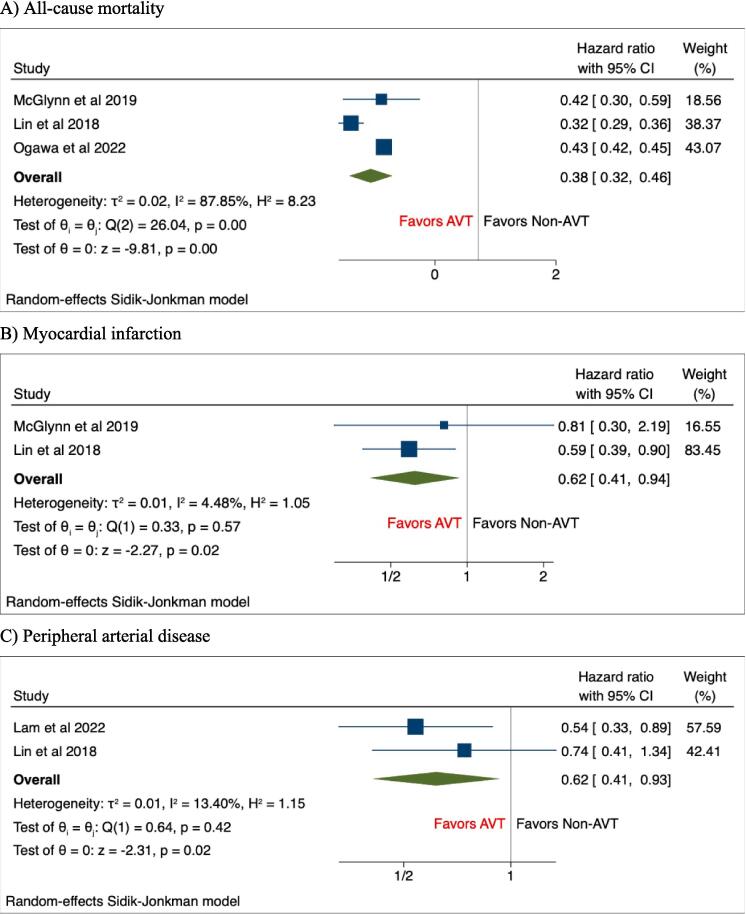
Forest plots of secondary outcomes including A) All-cause mortality, B) Myocardial infarction, C) Peripheral arterial disease.

**Fig. 3:**
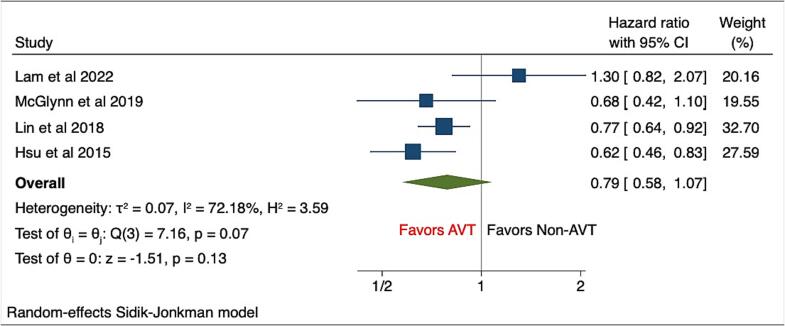
Forest plots of secondary outcome including stroke.

Secondary outcomes including the risk of ACM (HR, 0.38(95%CI: 0.32–0.46), P < 0.001, I^2^ = 87.85%), MI (HR, 0.62(95%CI: 0.41–0.94), P = 0.02, I^2^ = 4.48%), and PAD (HR, 0.62(95%CI: 0.41–0.93), P = 0.02, I^2^ = 13.40%) were significantly lower among AVT groups compared with NAVT groups among Hepatitis C infected patients **(**Fig. 2A-C**)**. However, the risk of stroke was comparable between both the groups of patients (HR, 0.79(95%CI: 0.58–1.07), P = 0.13, I^2^ = 72.18%) **(**Fig. 3**)**.

### 3.4 Sensitivity analysis

Sensitivity analyses were performed for any CVD, ACM, and stroke owing to high heterogeneity. Results of CVD and ACM remained significant and unaltered after sensitivity analysis by excluding one study at a time (p < 0.05) **(**Supplementary Fig. 2A-B**)**. After excluding the study by Lam et al, we observed that there was a statistically significant lower risk of stroke among the AVT group compared to the NAVT group (HR, 0.71(95%CI: 0.60–0.85), P < 0.001), which is contrary to the primary result and was suggestive of potential influence of study by Lam et al on the effect size on the risk of stroke **(**Supplementary Fig. 3**)**. Concurrently, it was noted that heterogeneity substantially reduced from 72.18% to 15.30% **(**Supplementary Fig. 4).

### 3.5 Subgroup analysis

Subgroup analyses were performed based on the type of antiviral therapy including directly acting antiviral (DAA) and interferon-based therapy (IBT). Results of subgroup analysis showed that there was no significant subgroup difference between DAA and IBT on the risk of any CVD (p = 0.96). On the other hand, studies using IBT showed a reduction in the risk of stroke compared to Non-AVT (HR, 0.71(95%CI: 0.58–0.88)) while there was no significant difference among studies using DAA therapy compared to Non-AVT (HR, 0.94(95%CI: 0.51–0.73)). However, there was no significant subgroup difference (p = 0.39) **(**Supplementary Figure 5**A-B)**.

### 3.6 Publication bias and quality assessment

The Funnel plot appeared asymmetrical for any CVD **(**Supplementary Figure 6**)**. However, the risk of any CVD remained significantly lower among AVT groups compared to NAVT groups after adjustment using the trim-and-fill method **(**Supplementary Figure 7**)**. There was no evidence of publication bias for stroke **(**Supplementary Figure 8**)**. The quality assessment using NOS for observational studies showed that there was a low risk of bias across studies **(**Supplementary Table 1).

## 4 Discussion

**Fig. 4:**
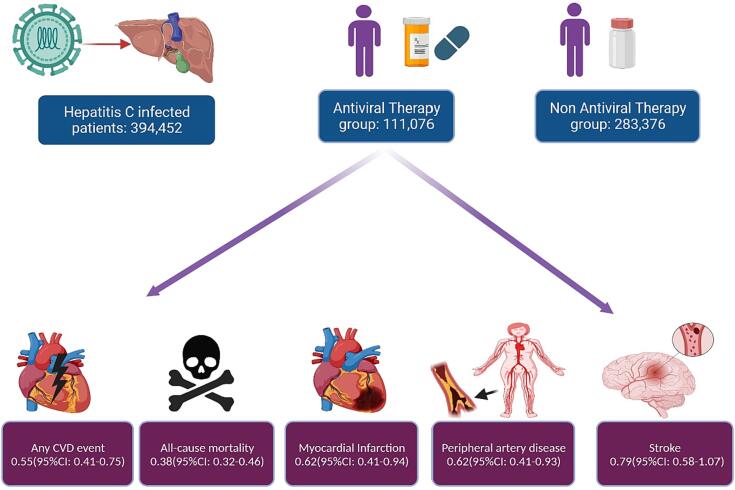
Central illustration highlighting the cardioprotective effect of antiviral therapy among Hepatitis C-infected patients.

In this meta-analysis, we evaluated the post-AVT cardiovascular outcomes in hepatitis C patients. In our study, we revealed that AVT significantly reduces our primary outcomes i.e., any cardiovascular disease in comparison to the NAVT group. Secondary outcomes i.e., myocardial infarction, all-cause mortality, and PAD were also significantly lower in the AVT group as compared to NAVT (Fig. 4). However, the risk of heart failure and stroke were comparable between the two groups i.e., AVT and NAVT. On the contrary, the effect of AVT on the reduction of strokes becomes significantly lower as compared to NAVT after removing Lam et al., showing the impact of this study on the risk of stroke in the overall population size (**46**). Similarly, on subgroup analysis, it was found that IBT significantly reduces stroke as compared to control.

A study conducted by Adinofil LE et al., to evaluate the impact of hepatitis C virus clearance by direct-acting AVT (DAA) on the incidence of cardiovascular events revealed that CV risk was two to three times lower in HCV-treated patients compared to control, with annual CV incident risk reduction by 68%, findings concordant with our results [29]. Similar CV reductions were found in direct-acting antiviral-treated hepatitis C patients in comparison to control, in studies conducted by Lam L et al., Butt AA et al., and Sasso FC et al., further supporting our findings [27], [31], [46]. Long-term mortality and safety in chronic hepatitis C patients were assessed by Ogawa E et al., and Mcglynn EA et al., and revealed that DAA-treated hepatitis C patients had significantly lower all-cause mortality as compared to non-treated control, results concordant with our findings [43], [41]. However, on subgroup analysis, contrary to a previous study by Butt et al., our study didn't find any significant difference in the reduction of CVD in the IBT group as compared to the control [27]. The secondary outcomes of our study i.e., MI, stroke, heart failure, and PAD were studied in a nationwide cohort study by Lin MS et al. and showed significantly reduced MI events in the DAA-treated group, results concordant with our findings while non-significant reduced PAD and significantly reduced stroke and heart failure in DAA treated group as compared to non-treated control, results discordant with our findings [45].

The pathogenesis of hepatitis C virus infection causing atherosclerosis and leading to increased cardiovascular events could be multifactorial and includes endothelial dysfunction, metabolic disturbances, lipid disturbances, oxidative stress, cytokine activation, and vascular injury [47], [48]. The Hepatitis C virus has been found to increase the risk of DM, a risk factor for CVD [49]. Additionally, increased inflammatory biomarkers have been found in HCV, a potential cause of increased cardiovascular events in these patients [50], [51]. A retrospective study conducted by Guzmán-Fulgencio M et al. revealed that AVT in HCV infection reduces inflammatory markers and improves endothelial function [52]. Improvement of endothelial function and reduction of oxidative stress state has been found in the literature after AVT. The mechanism might be involved in the improvement of atherosclerosis and reduction of cardiovascular events [53], [54]. Additionally, studies have revealed that AVT in HCV infection has a significant role in the reduction of fasting glucose as well as glycated hemoglobin, leading to reduced cardiovascular events [55], [56]. In conclusion, these studies proved that AVT in hepatitis C patients reduces cardiovascular events by improving endothelial function, reducing inflammatory biomarkers and oxidative stress state, and improving glycated hemoglobin.

AVT in hepatitis C patients is not only beneficial in the reduction of cardiovascular events and ACM but also has favorable results in the reduction of other complications of hepatitis C virus infection i.e., mixed cryoglobulinemia, porphyria cutanea tarda, glomerulonephritis and other associated pathologies like end-stage renal diseases, retinopathy, and non-Hodgkin lymphoma. The effect of AVT on extrahepatic manifestations of hepatitis C virus infection was studied by Mahale P et al. and revealed that AVT significantly reduces mixed cryoglobulinemia, porphyria cutanea tarda, glomerulonephritis, and non-Hodgkin lymphoma in comparison to the NAVT control group and these events were found more reduced in those patients who achieved sustained virological response (SVR) [30]. Similarly, significantly reduced end-stage renal diseases were reported in the AVT group compared to NAVT as well as in SVR-achieved hepatitis C patients in comparison to non-SVR-achieved hepatitis C patients in the literature [57], [58].

## 5 Limitations

The major limitation of the current study is that the results were largely derived from observational studies, where confounding bias could not be ruled out. Secondly, the number of studies was limited, thus precluding the ability to perform further analysis including meta-regression to evaluate potential effect modifiers. Furthermore, outcomes such as stroke were inconclusive and required further validation given that we identified the potential influence of a single study on the overall effect size. Sensitivity analysis confirmed that excluding that study resulted in a significant effect size in terms of the association between use of antivirals and reduction in the risk of stroke.

## 6 Conclusion

Our analysis shows HCV-infected patients post-AVT have a significantly lower risk of any CVD, MI, ACM, and PAD compared with NAVT groups of patients.

## 7 Ethical approval

Since this is a review article of previous published studies, ethical approval **i**s not required.

## 8 Author's Contribution

V.J. **contributed to the conception or design of the work.** S.A. V.J, **contributed to the acquisition, analysis, or interpretation of data for the work.** All authors help in **drafting the manuscript.** V.J, MH, and SPA. **critically revised the manuscript.** All gave final approval and agreed to be accountable for all aspects of work, ensuring integrity and accuracy.

## 9 Source of funding

None disclosed by authors.

## 10 Prospero registration number

CRD42023410973

## 11 Acknowledgment of Grant support for Funding

None disclosed by authors.

## Declaration of Competing Interest

The authors declare that they have no known competing financial interests or personal relationships that could have appeared to influence the work reported in this paper.

## Data Availability

The data underlying this article are available in the article and its online supplementary material.
