# Supplementary material for: Cardioprotective effect of antiviral therapy among hepatitis C infected patients: A meta-analysis
Source: Int J Cardiol Heart Vasc. 2023 Sep 22;49:101270. doi: 10.1016/j.ijcha.2023.101270 (PMC10520301; doi:10.1016/j.ijcha.2023.101270)
Supplement: Supplementary data 1 [file mmc1.docx]

**Supplementary Table 1:** Newcastle-Ottawa scale for quality assessment and bias assessment of observational studies.

**Supplementary Figure 1**: The Preferred Reporting Items for Systematic Reviews and Meta-Analyses (PRISMA) flow diagram

**Supplementary Figure 2** Leave-one-out analysis for outcomes including A) Any CVD, B) ACM

**Supplementary Figure 3** Leave-one-out analysis for Stroke

**Supplementary Figure 4** Sensitivity analysis for Stroke after excluding study by Lam et al.

**Supplementary Figure 5.** Subgroup analysis based on antiviral therapy type for A) CVD, and B) Stroke.

**Supplementary Figure 6** Funnel plot for Any CVD

**Supplementary Figure 7** Funnel plot for Any CVD after trim-and-fill method

**Supplementary Figure 8** Funnel plot for Stroke

**Supplementary Table 1:** Newcastle-Ottawa scale for quality assessment and bias assessment of observational studies.

| Study | Selection | | | | Comparability | Outcome | | | Total* |
| --- | --- | --- | --- | --- | --- | --- | --- | --- | --- |
| Author, year | Representatives of exposed group | Selection of non-exposed cohort | Ascertainment of exposure | Outcome of interest |  | Outcome assessment | Adequacy of follow up duration | Adequacy of follow up of cohort |  |
| Lam et al | 1 | 1 | 1 | 1 | 2 | 1 | 1 | 1 | 9 |
| McGlynn et al | 1 | 1 | 1 | 1 | 2 | 1 | 1 | 0 | 8 |
| Butt et al | 1 | 1 | 1 | 1 | 2 | 1 | 0 | 0 | 7 |
| Adinolfi et al | 1 | 1 | 1 | 1 | 2 | 1 | 1 | 0 | 9 |
| Lin et al | 1 | 1 | 1 | 1 | 2 | 1 | 1 | 1 | 9 |
| Sasso et al | 1 | 1 | 1 | 1 | 1 | 1 | 1 | 1 | 8 |
| Hsu et al | 1 | 1 | 1 | 1 | 1 | 1 | 1 | 1 | 8 |
| Ogawa et al | 1 | 1 | 1 | 1 | 1 | 1 | 0 | 0 | 6 |


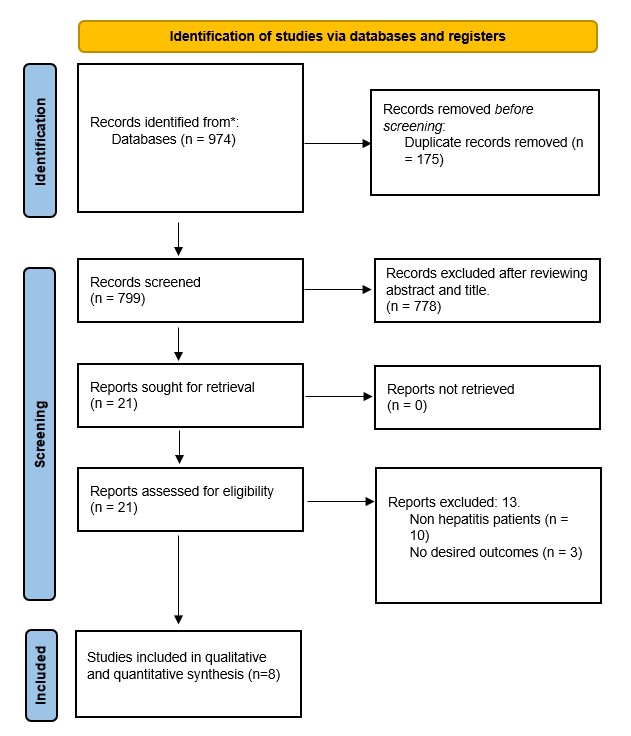


**Supplementary Figure 1**: The Preferred Reporting Items for Systematic Reviews and Meta-Analyses (PRISMA) flow diagram

A) Any CVD

**
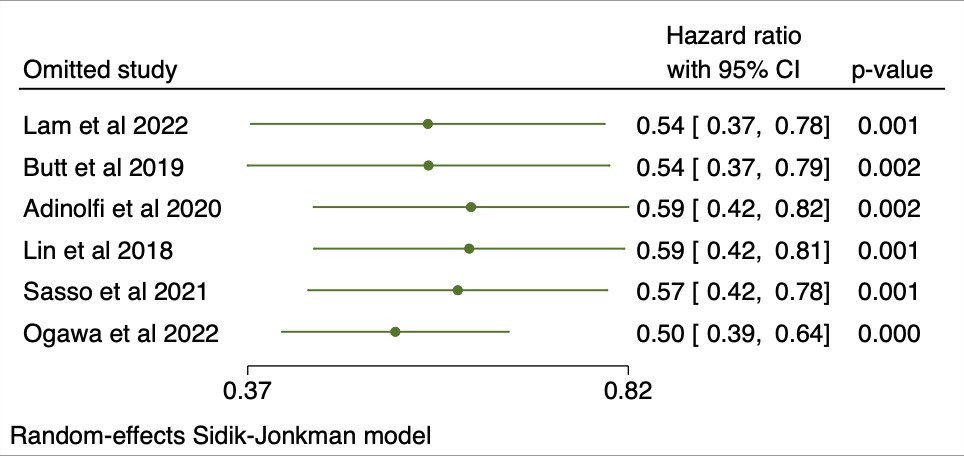
**

B) ACM

**
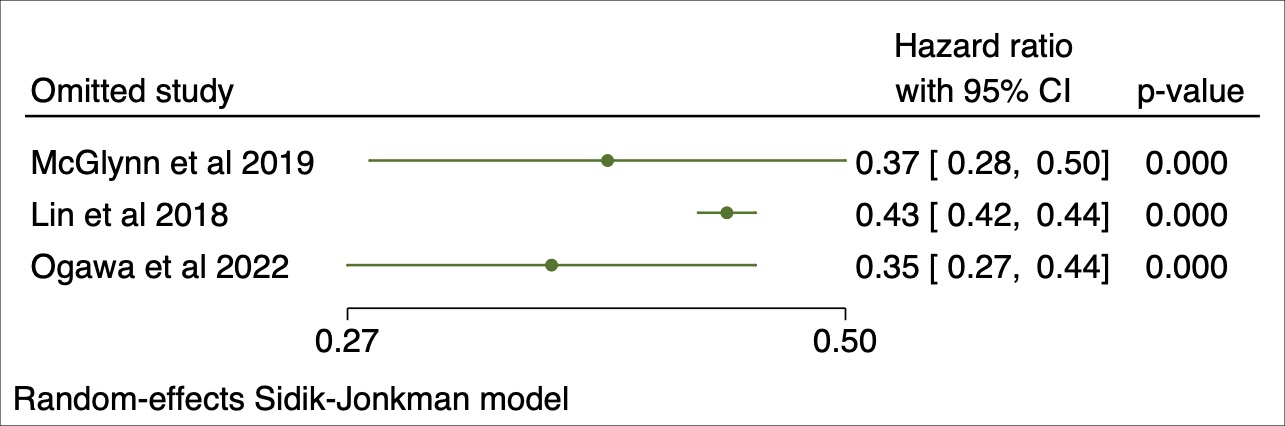
**

**Supplementary Figure 2** Leave-one-out analysis for outcomes including A) Any CVD, B) ACM

**
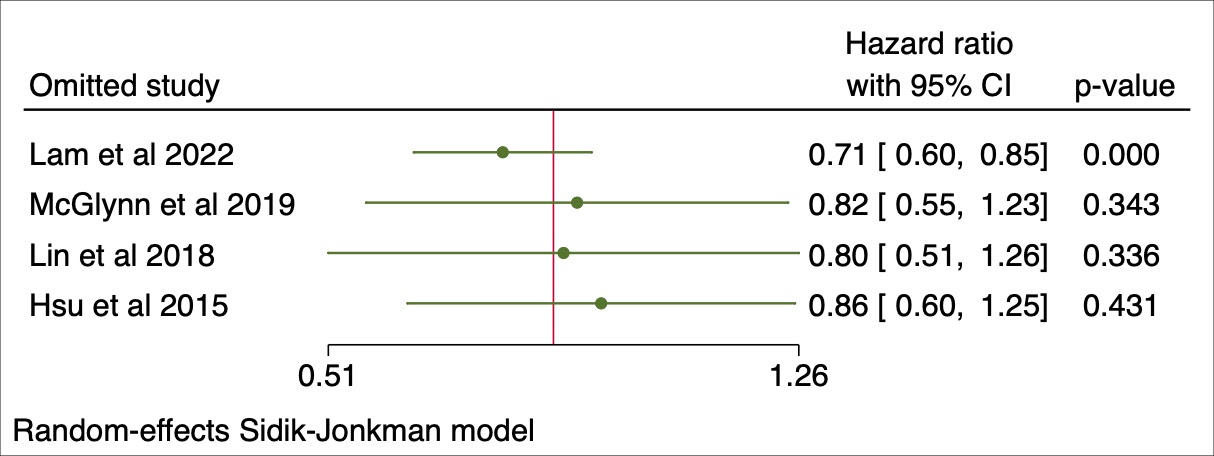
**

**Supplementary Figure 3** Leave-one-out analysis for Stroke


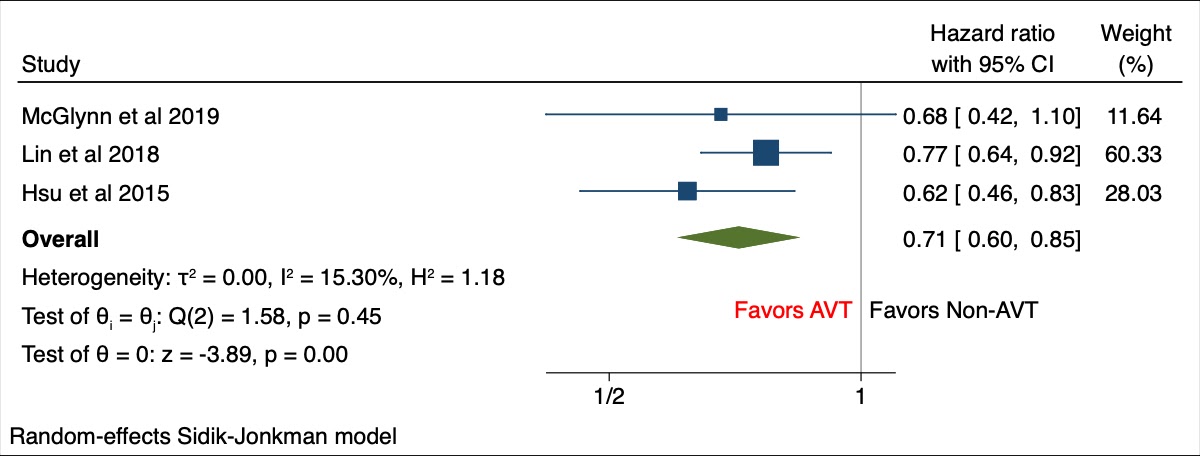


**Supplementary Figure 4** Sensitivity analysis for Stroke after excluding study by Lam et al

1. CVD


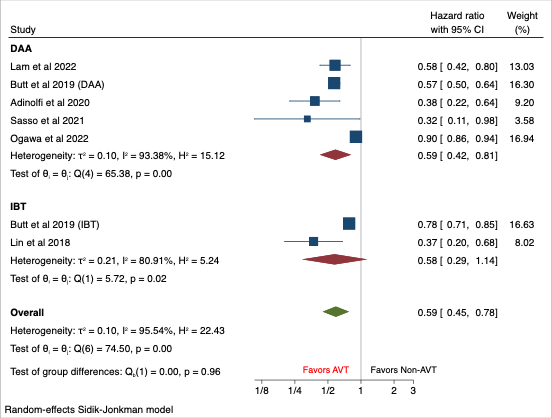


1. Stroke


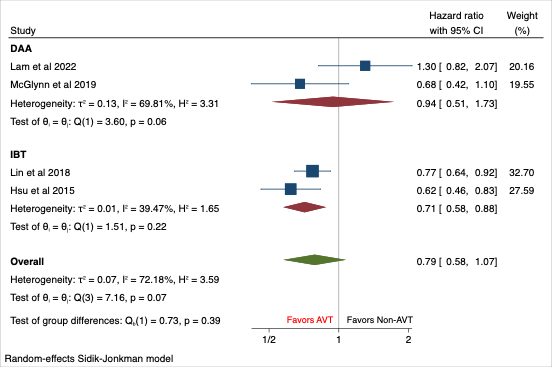
**Supplementary Figure 5.** Subgroup analysis based on antiviral therapy type for A) CVD, and B) Stroke.

**
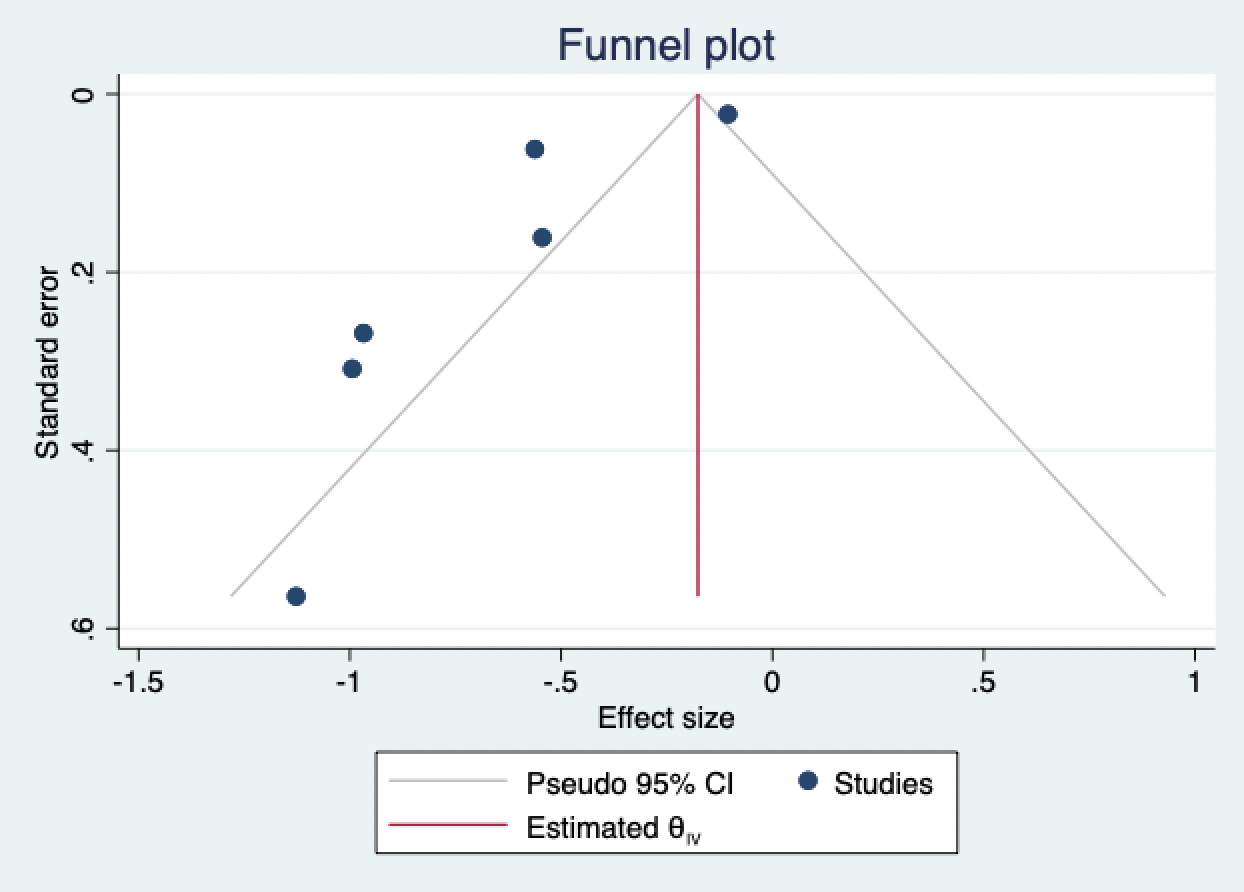
**

**Supplementary Figure 6.** Funnel plot for Any CVD

**
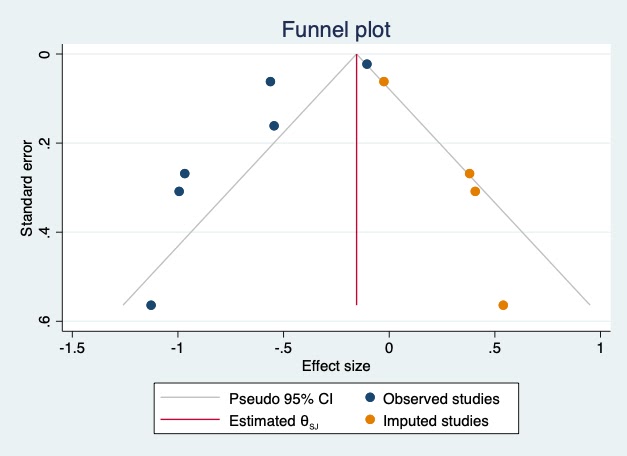
**

**Supplementary Figure 7.** Funnel plot for Any CVD after trim-and-fill method

**
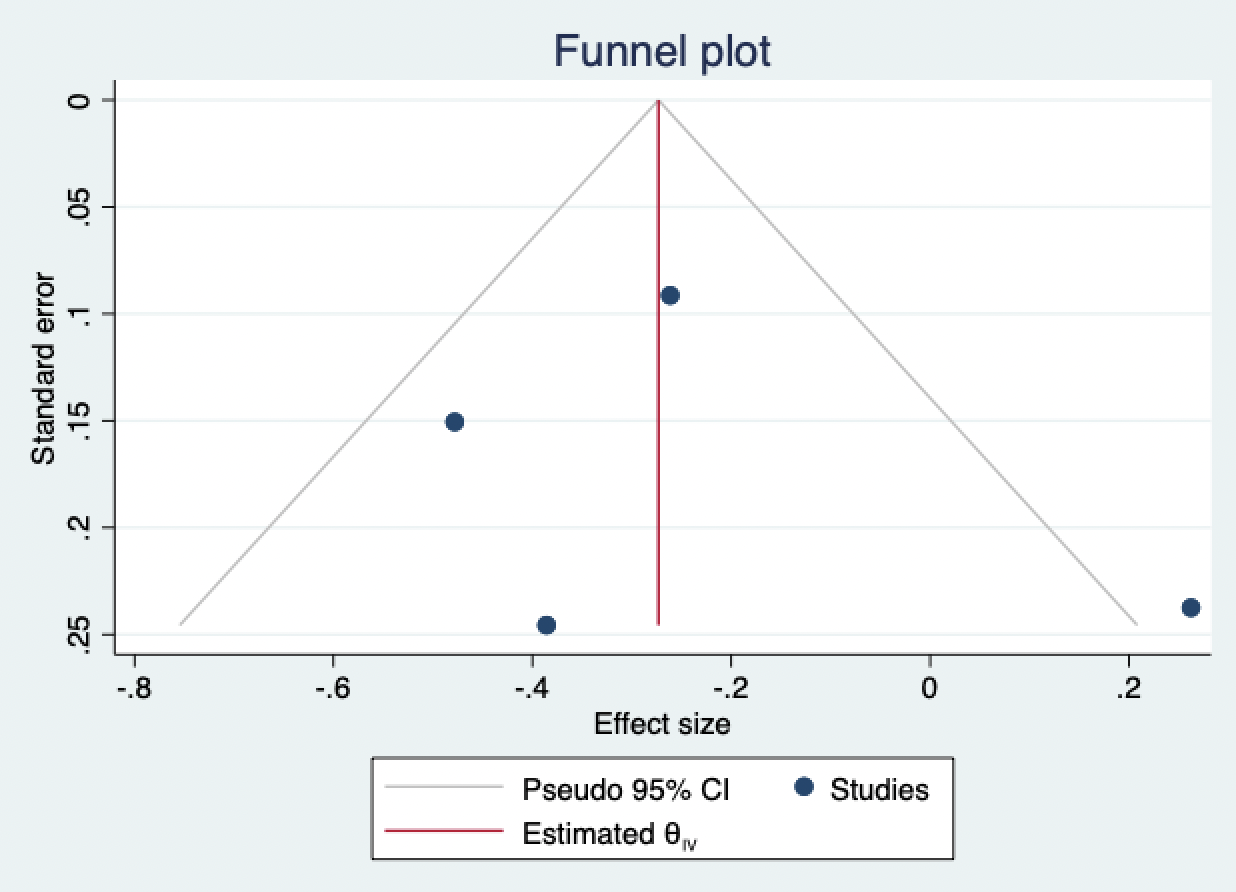
**

**Supplementary Figure 8.** Funnel plot for Stroke
